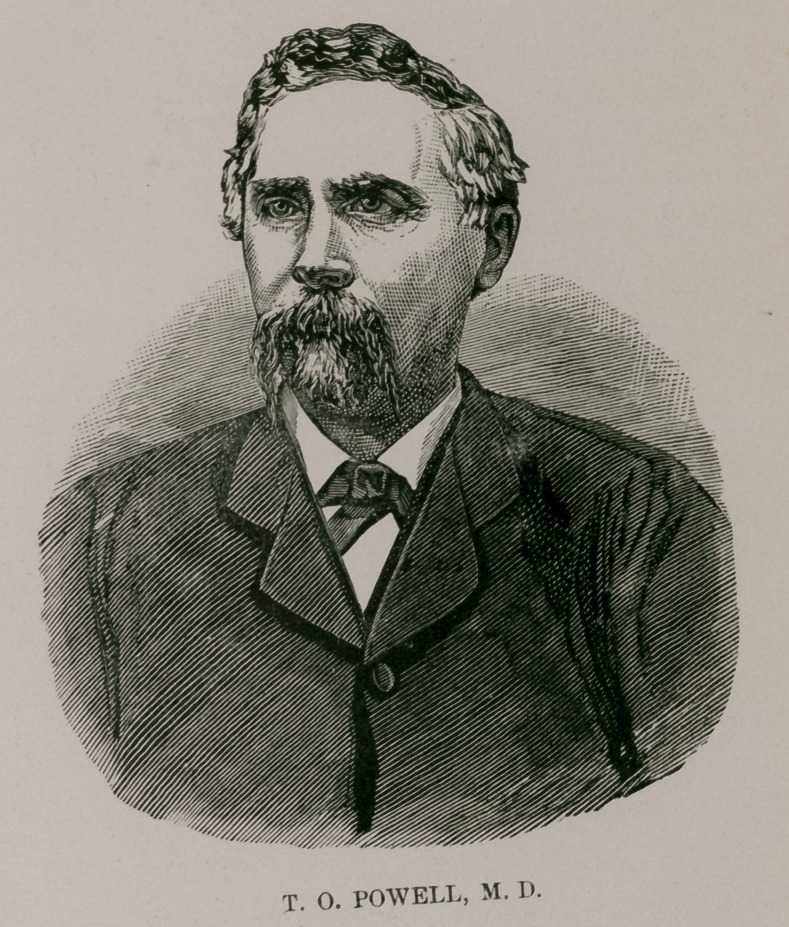# Our Portrait Gallery

**Published:** 1885-04

**Authors:** 


### OUR PORTRAIT GALLERY.

### T. O. POWELL, M. D.

The subject of this sketch was born in Virginia in 1837. His parents removed to Hancock county, Georgia, while he was yet a child. Here he was brought up, and attended his first school. After spending a few years in the schools in Sparta, he was sent

T. O. POWELL, M. D.

to Petersburg, Virginia, where he finished his literary education. Returning from Petersburg in 1854, he began the study of medicine, and entered the Medical College of Georgia (now the Medical Department of the University of Georgia), in 1856 and graduated therefrom in 1858. The spring following his graduation he began the practice of medicine in Sparta, Georgia. Here he made an enviable reputation as a successful practitioner.

Dr. Powell continued to practice in Sparta until August, 1862, when he was elected first assistant physician to the Georgia State Lunatic Asylum. Soon after his election to this responsible place he entered upon the duties of the office which he filled with great credit to himself until the death of the Principal and Superintendent, Dr. Green, in 1879, when he was elected to this position, which he continues to fill with credit to himself and satisfaction to the Board of Trustees and the people of Georgia.

Dr. Powell's gentle, kind and amiable disposition, coupled with his good judgment and eminent ability, renders him particularly fit to preside over such an institution. He has bestowed a great deal of time and attention to the sanitary arrangement of the buildings, and we doubt not that the methods of ventilation adopted by him are superior to those of any similar institution in the land.

His kindness and care of the unfortunate inmates of the asylum have won for him the love and respect of every one.

In 1867 Dr. Powell was elected a member of the Medical Association of Georgia, and has made frequent contributions to the annual meeting of this body. Among his valuable contributions to medical literature may be mentioned one of especial value: Hyoscyamin in Violent Mania, which was published in the Transactions of the Medical Association of Georgia in 1883.

Dr. Powell was married in 1860 to Miss Fannie Birdsong, daughter of Edward Birdsong, of Hancock, Georgia. She is most elegant lady, with many rare and lovely traits of character. She has proved a devoted wife, has blessed his life and rejoiced with him in his success.

## Figures and Tables

**Figure f1:**